# The genome sequence of the Four-banded Bee-grabber, *Conops quadrifasciatus *De Geer, 1776

**DOI:** 10.12688/wellcomeopenres.21106.1

**Published:** 2024-03-08

**Authors:** Steven Falk, Liam M. Crowley, David K. Clements, University of Oxford and Wytham Woods Genome Acquisition Lab, Darwin Tree of Life Barcoding collective, Wellcome Sanger Institute Tree of Life Management, Samples and Laboratory team, Wellcome Sanger Institute Scientific Operations: Sequencing Operations, Wellcome Sanger Institute Tree of Life Core Informatics team, Tree of Life Core Informatics collective, Darwin Tree of Life Consortium

**Affiliations:** 1 Independent researcher, Kenilworth, England, UK; 2 University of Oxford, Oxford, England, UK; 3 Independent researcher, Cardiff, Wales, UK

**Keywords:** Conops quadrifasciatus, Four-banded Bee-grabber, genome sequence, chromosomal, Diptera

## Abstract

We present a genome assembly from an individual male *Conops quadrifasciatus* (the Four-banded Bee-grabber; Arthropoda; Insecta; Diptera; Conopidae). The genome sequence is 210.4 megabases in span. Most of the assembly is scaffolded into 7 chromosomal pseudomolecules, including the X and Y sex chromosomes. The mitochondrial genome has also been assembled and is 18.07 kilobases in length. Gene annotation of this assembly on Ensembl identified 23,090 protein coding genes.

## Species taxonomy

Eukaryota; Opisthokonta; Metazoa; Eumetazoa; Bilateria; Protostomia; Ecdysozoa; Panarthropoda; Arthropoda; Mandibulata; Pancrustacea; Hexapoda; Insecta; Dicondylia; Pterygota; Neoptera; Endopterygota; Diptera; Brachycera; Muscomorpha; Eremoneura; Cyclorrhapha; Schizophora; Acalyptratae; Conopoidea; Conopidae; Conopinae; *Conops*; *Conops quadrifasciatus* De Geer, 1776 (NCBI:txid2823188).

## Background

*Conops quadrifasciatus* is a member of the acalyptrate family Conopidae within the Diptera, or ‘true flies’. As far as is known, all members of this family develop as endoparasitoids in other insects, chiefly the adults of aculeate Hymenoptera ( Stuke, 2016).

*Conops quadrifasciatus* is a widespread and common Palaearctic species which occurs in most countries of Europe as well as in Russia, Ukraine, Türkiye and Iran, extending as far east as Kyrgyzstan and Novosibirsk ( GBIF Secretariat, 2024; iNaturalist, 2024; Stuke, 2016). The adult is one of several rather similar black and yellow species of rather elongated, wasp-like appearance, which probably benefit from mimicry of aposematic Hymenoptera. The nectar-feeding adults are chiefly found in warm, sunlit and flower-rich habitats, often on umbels and other flower-heads amongst swarms of similar-looking insects including various hoverflies, bees and wasps.

Females of *C. quadrifasciatus* lay their eggs directly into the abdominal cavities of adult bees, primarily members of the bumblebee genus *Bombus* ( Stuke, 2016). Host bees are usually intercepted in flight, the conopid grabbing the bee with its long legs and wrestling with it while tumbling to the ground. Female conopids are characterised by specialised adaptations of the abdomen which create a pincer-like mechanism formed by the elongated final segment of the abdomen and a corresponding bulge on the underside of the fifth segment called the ‘theca’. This mechanism is probably used to pry apart the abdominal segments of the host to allow an egg to be inserted through the intersegmental membrane ( Clements, 1997). Oviposition usually occurs very rapidly, however, and the mechanics of it are still not well understood.

Within a few days the conopid egg hatches and the larva attaches itself to the host’s tracheal system for oxygenation. The larva initially devours the host’s fat-bodies and other non-essential organs. Usually there is only one conopid larva per host. The depredations of the conopid larva make the host increasingly unable to function normally leading to alterations in its behaviour, for example by focusing only on easily foraged flowers or undertaking shorter foraging journeys ( Schmid-Hempel, 1998; Schmid-Hempel & Schmid-Hempel, 1990). Infested hosts may also remain out of the nest at night, which has been shown to slow down the development of the conopid larva through exposure to lower temperatures. In some cases, this may allow the host to complete most of its natural lifespan before the conopid larva is able to complete its development. In many cases, however, the conopid larva ultimately kills the host ( Clements, 1997; Schmid-Hempel, 1998).

In its final instar the conopid larva develops a long, attenuated anterior section with the mouth at its tip. Having consumed the abdominal contents, the larva probes through the host’s narrow petiole to consume the thoracic organs, causing its death. Prior to host death, the conopid will often invoke fossorial behaviour, causing the host to dig itself into the soil before it dies. The conopid larva then pupates in the buried husk where it is protected from adverse weather and predation by scavengers ( Müller, 1994). After overwintering, the adult conopid hatches and digs its way to the surface.

Whilst adult conopids tend to be found only at relatively low densities, research has shown that larval infestation in host bees can occur at rates of 30–70% ( Schmid-Hempel, 1998). Conopids are therefore considered to represent potentially significant predators of bees and wasps, with some species being significant pests in commercial honey production worldwide ( Tingek *et al.*, 2007).

Conopid species are taxonomically complex with many forms of uncertain taxonomic status, including within the genus *Conops*. The whole genome sequence present here may assist in resolving these taxonomic uncertainties ( Stuke, 2016).

## Genome sequence report

**Figure 1.**
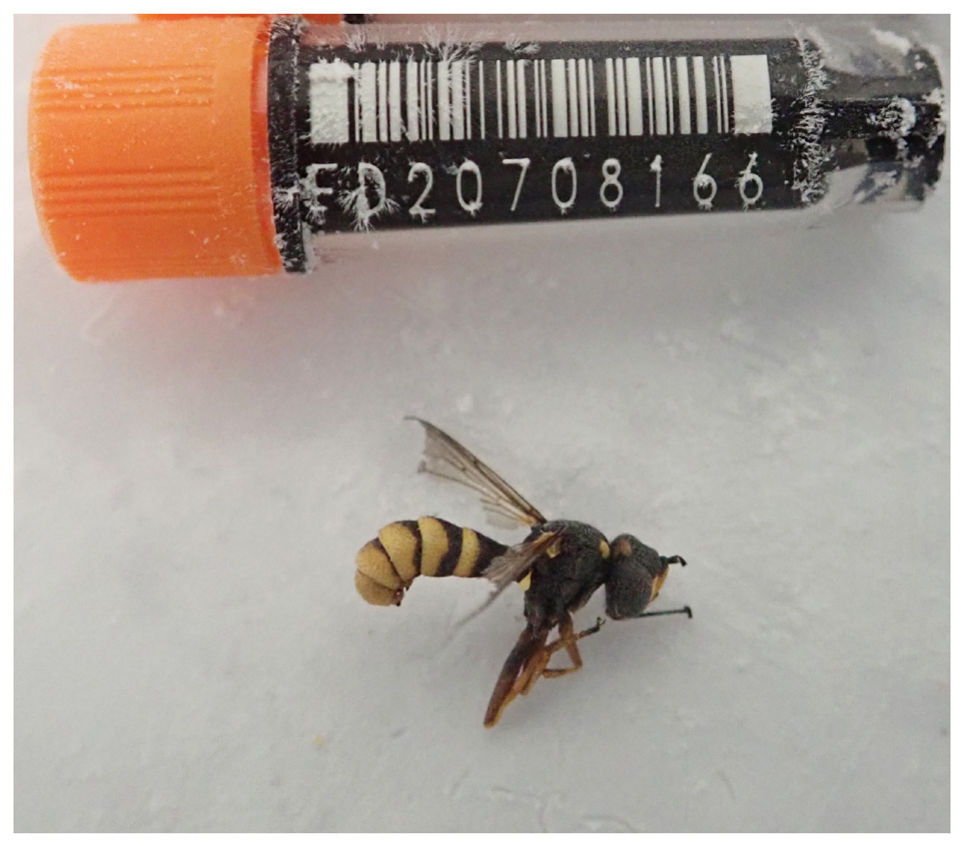
Photograph of the *Conops quadrifasciatus* (idConQuad1) specimen used for genome sequencing.

The genome was sequenced from one specimen of *Conops quadrifasciatus* ( Figure 1) collected from Wytham Woods, Oxfordshire, UK (51.77, –1.31). A total of 95-fold coverage in Pacific Biosciences single-molecule HiFi long reads was generated. Primary assembly contigs were scaffolded with chromosome conformation Hi-C data. Manual assembly curation corrected 37 missing joins or mis-joins and removed 8 haplotypic duplications, reducing the assembly length by 0.12% and the scaffold number by 12.50%, and increasing the scaffold N50 by 15.24%.

**Figure 2.**
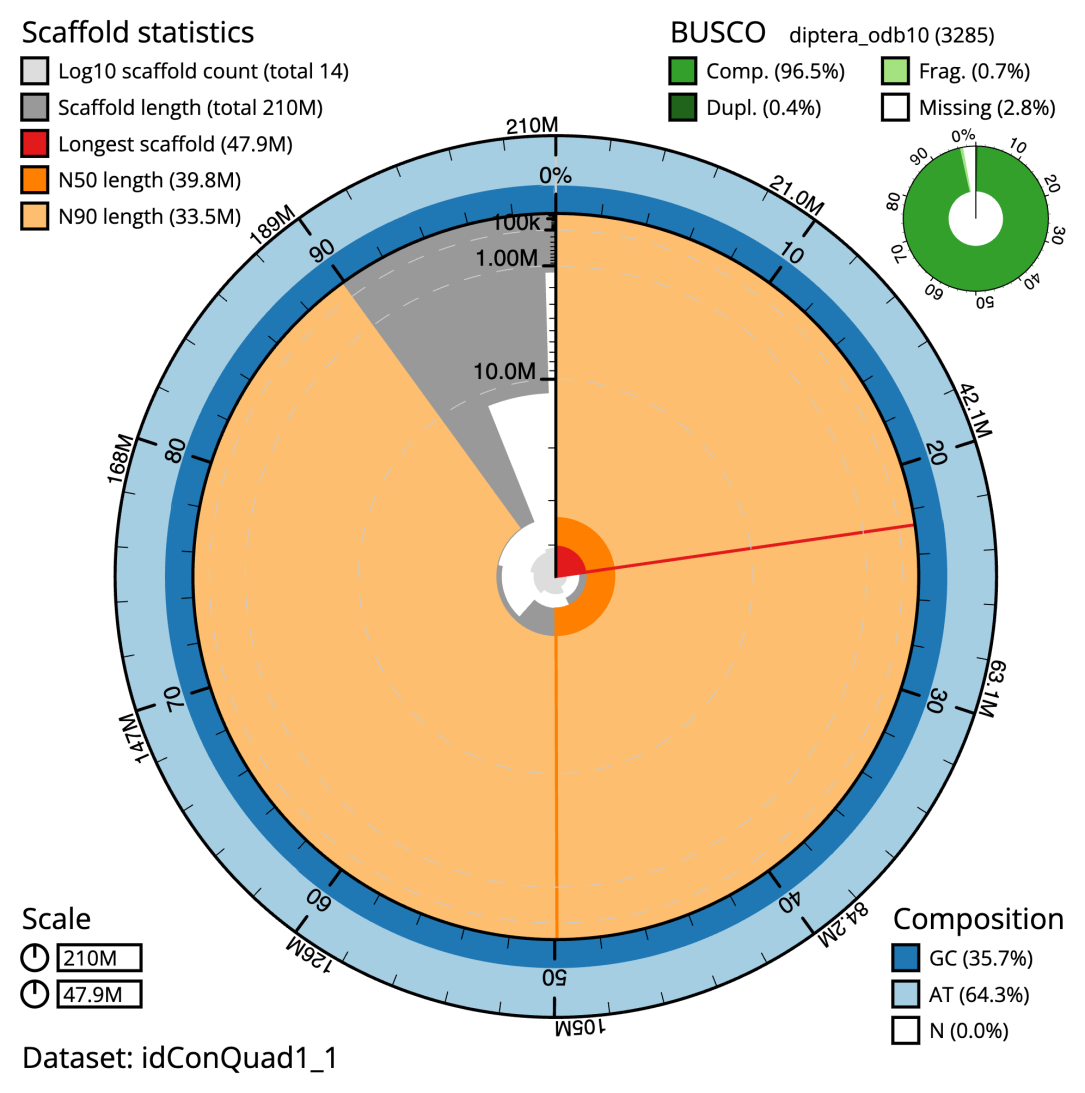
Genome assembly of *Conops quadrifasciatus*, idConQuad1.1: metrics. The BlobToolKit snail plot shows N50 metrics and BUSCO gene completeness. The main plot is divided into 1,000 size-ordered bins around the circumference with each bin representing 0.1% of the 210,459,157 bp assembly. The distribution of scaffold lengths is shown in dark grey with the plot radius scaled to the longest scaffold present in the assembly (47,946,236 bp, shown in red). Orange and pale-orange arcs show the N50 and N90 scaffold lengths (39,758,196 and 33,513,057 bp), respectively. The pale grey spiral shows the cumulative scaffold count on a log scale with white scale lines showing successive orders of magnitude. The blue and pale-blue area around the outside of the plot shows the distribution of GC, AT and N percentages in the same bins as the inner plot. A summary of complete, fragmented, duplicated and missing BUSCO genes in the diptera_odb10 set is shown in the top right. An interactive version of this figure is available at https://blobtoolkit.genomehubs.org/view/idConQuad1_1/dataset/idConQuad1_1/snail.

**Figure 3.**
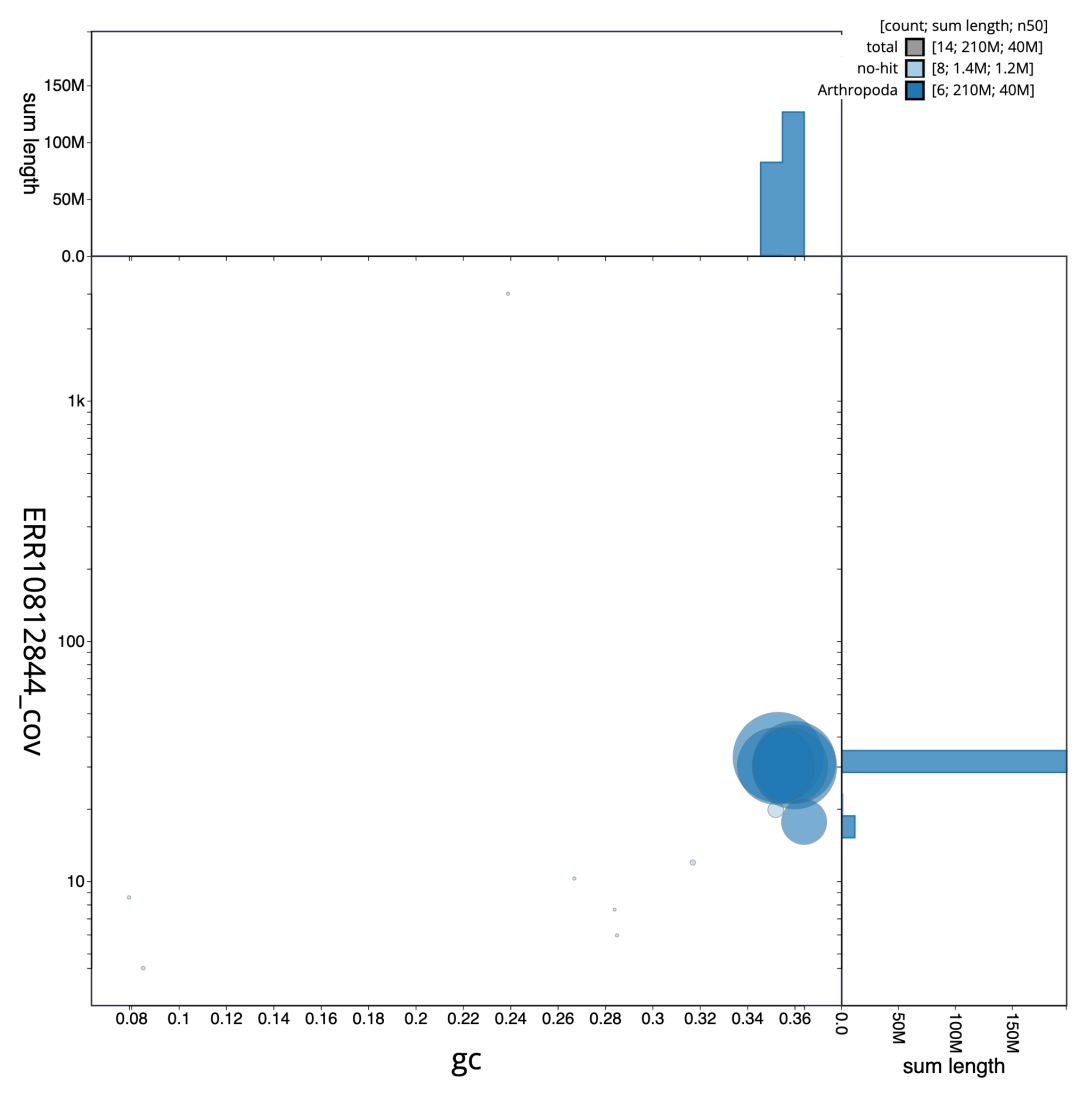
Genome assembly of *Conops quadrifasciatus*, idConQuad1.1: BlobToolKit GC-coverage plot. Sequences are coloured by phylum. Circles are sized in proportion to sequence length. Histograms show the distribution of sequence length sum along each axis. An interactive version of this figure is available at https://blobtoolkit.genomehubs.org/view/idConQuad1_1/dataset/idConQuad1_1/blob.

**Figure 4.**
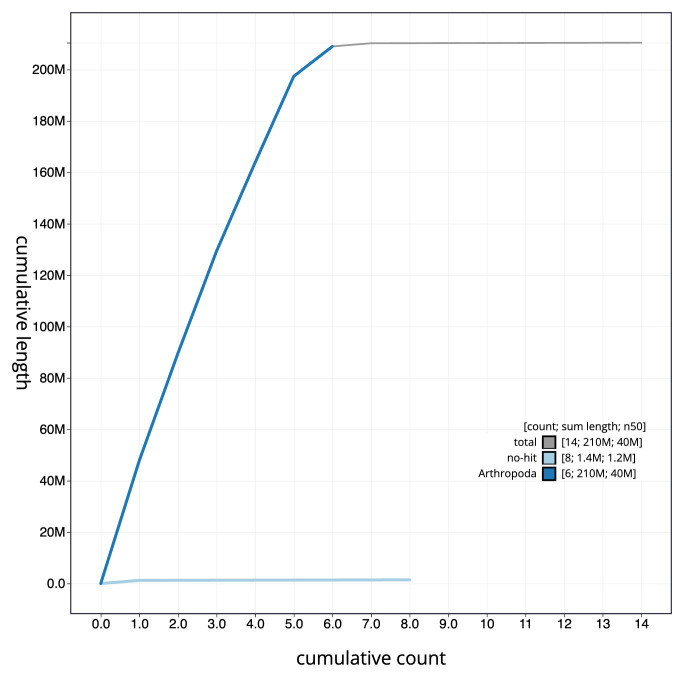
Genome assembly of *Conops quadrifasciatus*, idConQuad1.1: BlobToolKit cumulative sequence plot. The grey line shows cumulative length for all sequences. Coloured lines show cumulative lengths of sequences assigned to each phylum using the buscogenes taxrule. An interactive version of this figure is available at https://blobtoolkit.genomehubs.org/view/idConQuad1_1/dataset/idConQuad1_1/cumulative.

**Figure 5.**
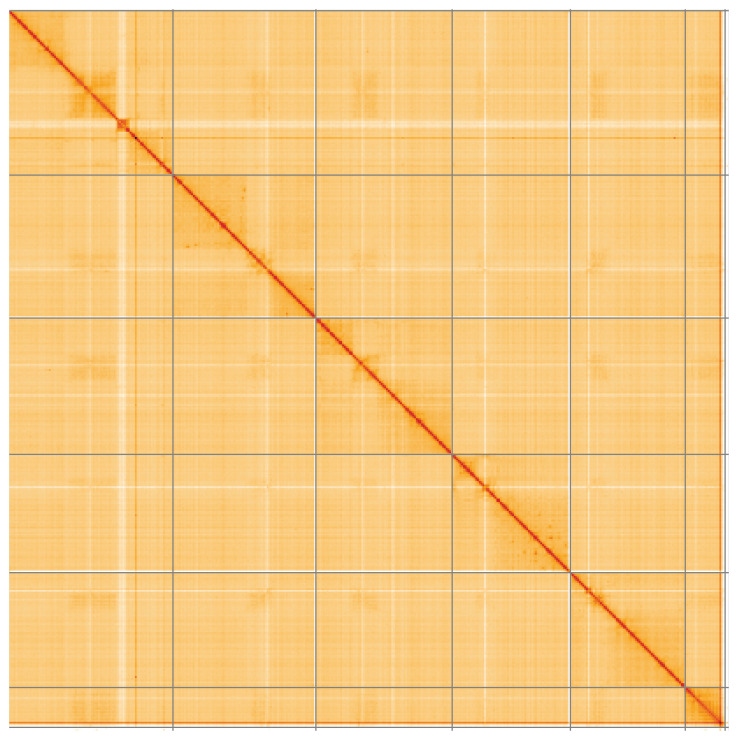
Genome assembly of *Conops quadrifasciatus*, idConQuad1.1: Hi-C contact map of the idConQuad1.1 assembly, visualised using HiGlass. Chromosomes are shown in order of size from left to right and top to bottom. An interactive version of this figure may be viewed at https://genome-note-higlass.tol.sanger.ac.uk/l/?d=IgeuY3TgSre9iwe0t-9iUg.

The final assembly has a total length of 210.4 Mb in 13 sequence scaffolds with a scaffold N50 of 39.8 Mb ( Table 1). The snail plot in Figure 2 provides a summary of the assembly statistics, while the distribution of assembly scaffolds on GC proportion and coverage is shown in Figure 3. The cumulative assembly plot in Figure 4 shows curves for subsets of scaffolds assigned to different phyla. Most (99.96%) of the assembly sequence was assigned to 7 chromosomal-level scaffolds, representing 5 autosomes and the X and Y sex chromosomes. Chromosome-scale scaffolds confirmed by the Hi-C data are named in order of size ( Figure 5; Table 2). The sex chromosomes identified using coverage information. Chromosome Y scaffold was determined by mapping Hi-C data from a female sample to the male assembly. While not fully phased, the assembly deposited is of one haplotype. Contigs corresponding to the second haplotype have also been deposited. The mitochondrial genome was also assembled and can be found as a contig within the multifasta file of the genome submission.

**Table 1.** Genome data for *Conops quadrifasciatus*, idConQuad1.1.

Project accession data		
Assembly identifier	idConQuad1.1	
Species	*Conops quadrifasciatus*	
Specimen	idConQuad1	
NCBI taxonomy ID	2823188	
BioProject	PRJEB59143	
BioSample ID	SAMEA8603151	
Isolate information	idConQuad1, male: thorax (DNA sequencing) idConQuad2, female: head and thorax (Hi-C and RNA sequencing)	
Assembly metrics *		*Benchmark*
Consensus quality (QV)	63.6	*≥ 50*
*k*-mer completeness	100.0%	*≥ 95%*
BUSCO **	C:96.5%[S:96.1%,D:0.4%],F:0.7%,M:2.8%,n:3,285	*C ≥ 95%*
Percentage of assembly mapped to chromosomes	99.96%	*≥ 95%*
Sex chromosomes	XY	*localised homologous pairs*
Organelles	Mitochondrial genome: 18.07 kb	*complete single alleles*
Raw data accessions		
PacificBiosciences SEQUEL II	ERR10812844	
Hi-C Illumina	ERR10802472	
PolyA RNA-Seq Illumina	ERR11837463	
Genome assembly		
Assembly accession	GCA_949752815.1	
*Accession of alternate haplotype*	GCA_949752755.1	
Span (Mb)	210.4	
Number of contigs	48	
Contig N50 length (Mb)	13.2	
Number of scaffolds	13	
Scaffold N50 length (Mb)	39.8	
Longest scaffold (Mb)	47.95	
Genome annotation		
Number of protein-coding genes	23,090	
Number of gene transcripts	23,804	

* Assembly metric benchmarks are adapted from column VGP-2020 of “Table 1: Proposed standards and metrics for defining genome assembly quality” from Rhie *et al.* (2021). ** BUSCO scores based on the diptera_odb10 BUSCO set using version 5.3.2. C = complete [S = single copy, D = duplicated], F = fragmented, M = missing, n = number of orthologues in comparison. A full set of BUSCO scores is available at https://blobtoolkit.genomehubs.org/view/idConQuad1_1/dataset/idConQuad1_1/busco.

**Table 2.** Chromosomal pseudomolecules in the genome assembly of *Conops quadrifasciatus*, idConQuad1.

INSDC accession	Chromosome	Length (Mb)	GC%
OX457077.1	1	47.95	35.5
OX457078.1	2	41.7	36.0
OX457079.1	3	39.76	36.0
OX457080.1	4	34.49	35.0
OX457081.1	5	33.51	36.0
OX457082.1	X	11.62	36.5
OX457083.1	Y	1.23	35.0
OX457084.1	MT	0.02	24.5

The estimated Quality Value (QV) of the final assembly is 63.6 with *k*-mer completeness of 100.0%, and the assembly has a BUSCO v5.3.2 completeness of 96.5% (single = 96.1%, duplicated = 0.4%), using the diptera_odb10 reference set ( *n* = 3,285).

Metadata for specimens, barcode results, spectra estimates, sequencing runs, contaminants and pre-curation assembly statistics are given at https://links.tol.sanger.ac.uk/species/2823188.

## Genome annotation report

The *Conops quadrifasciatus* genome assembly (GCA_949752815.1) was annotated using the Ensembl rapid annotation pipeline at the European Bioinformatics Institute (EBI). The resulting annotation includes 23,804 transcribed mRNAs from 23,090 protein-coding genes. ( Table 1; https://rapid.ensembl.org/Conops_quadrifasciatus_GCA_949752815.1/Info/Index).

## Methods

### Sample acquisition and nucleic acid extraction

A male *Conops quadrifasciatus* (specimen ID Ox000913, ToLID idConQuad1) was netted at Wytham Woods, Oxfordshire (biological vice-county Berkshire), UK (latitude 51.77, longitude –1.31) on 2020-08-03. The specimen was collected and identified by Steven Falk (independent researcher). A male specimen was used for DNA sequencing, and a female specimen was used for Hi-C sequencing. A female specimen, used for Hi-C and RNA sequencing (specimen ID Ox001841, ToLID idConQuad2), was netted at the same location on 2021-08-21. This specimen was collected and identified by Liam Crowley (University of Oxford). Both specimens were snap-frozen on dry ice.

The workflow for high molecular weight (HMW) DNA extraction at the Wellcome Sanger Institute (WSI) includes a sequence of core procedures: sample preparation; sample homogenisation, DNA extraction, fragmentation, and clean-up. In sample preparation, the idConQuad1 sample was weighed and dissected on dry ice ( Jay *et al.*, 2023), and tissue from the thorax was homogenised using a PowerMasher II tissue disruptor ( Denton *et al.*, 2023a). HMW DNA was extracted using the Automated MagAttract v1 protocol ( Sheerin *et al.*, 2023). DNA was sheared into an average fragment size of 12–20 kb in a Megaruptor 3 system with speed setting 30 ( Todorovic *et al.*, 2023). Sheared DNA was purified by solid-phase reversible immobilisation ( Strickland *et al.*, 2023): in brief, the method employs a 1.8X ratio of AMPure PB beads to sample to eliminate shorter fragments and concentrate the DNA. The concentration of the sheared and purified DNA was assessed using a Nanodrop spectrophotometer and Qubit Fluorometer and Qubit dsDNA High Sensitivity Assay kit. Fragment size distribution was evaluated by running the sample on the FemtoPulse system.

RNA was extracted from head and thorax tissue of idConQuad1 in the Tree of Life Laboratory at the WSI using the RNA Extraction: Automated MagMax™ *mir*Vana protocol ( do Amaral *et al.*, 2023). The RNA concentration was assessed using a Nanodrop spectrophotometer and a Qubit Fluorometer using the Qubit RNA Broad-Range Assay kit. Analysis of the integrity of the RNA was done using the Agilent RNA 6000 Pico Kit and Eukaryotic Total RNA assay.

Protocols developed by the WSI Tree of Life laboratory are publicly available on protocols.io ( Denton *et al.*, 2023b).

### Sequencing

Pacific Biosciences HiFi circular consensus DNA sequencing libraries were constructed according to the manufacturers’ instructions. Poly(A) RNA-Seq libraries were constructed using the NEB Ultra II RNA Library Prep kit. DNA and RNA sequencing was performed by the Scientific Operations core at the WSI on Pacific Biosciences SEQUEL II (HiFi) and Illumina NovaSeq 6000 (RNA-Seq) instruments. Hi-C data were also generated from head and thorax tissue of idConQuad2 using the Arima2 kit and sequenced on the Illumina NovaSeq 6000 instrument.

### Genome assembly, curation and evaluation

Assembly was carried out with Hifiasm ( Cheng *et al.*, 2021) and haplotypic duplication was identified and removed with purge_dups ( Guan *et al.*, 2020). The assembly was then scaffolded with Hi-C data ( Rao *et al.*, 2014) using YaHS ( Zhou *et al.*, 2023). The assembly was checked for contamination and corrected as described previously ( Howe *et al.*, 2021). Manual curation was performed using HiGlass ( Kerpedjiev *et al.*, 2018) and PretextView ( Harry, 2022). The mitochondrial genome was assembled using MitoHiFi ( Uliano-Silva *et al.*, 2023), which runs MitoFinder ( Allio *et al.*, 2020) or MITOS ( Bernt *et al.*, 2013) and uses these annotations to select the final mitochondrial contig and to ensure the general quality of the sequence.

A Hi-C map for the final assembly was produced using bwa-mem2 ( Vasimuddin *et al.*, 2019) in the Cooler file format ( Abdennur & Mirny, 2020). To assess the assembly metrics, the *k*-mer completeness and QV consensus quality values were calculated in Merqury ( Rhie *et al.*, 2020). This work was done using Nextflow ( Di Tommaso *et al.*, 2017) DSL2 pipelines “sanger-tol/readmapping” ( Surana *et al.*, 2023a) and “sanger-tol/genomenote” ( Surana *et al.*, 2023b). The genome was analysed within the BlobToolKit environment ( Challis *et al.*, 2020) and BUSCO scores ( Manni *et al.*, 2021; Simão *et al.*, 2015) were calculated.

Table 3 contains a list of relevant software tool versions and sources.

**Table 3.** Software tools: versions and sources.

Software tool	Version	Source
BlobToolKit	4.2.1	https://github.com/blobtoolkit/blobtoolkit
BUSCO	5.3.2	https://gitlab.com/ezlab/busco
Hifiasm	0.16.1-r375	https://github.com/chhylp123/hifiasm
HiGlass	1.11.6	https://github.com/higlass/higlass
Merqury	MerquryFK	https://github.com/thegenemyers/MERQURY.FK
MitoHiFi	2	https://github.com/marcelauliano/MitoHiFi
PretextView	0.2	https://github.com/wtsi-hpag/PretextView
purge_dups	1.2.3	https://github.com/dfguan/purge_dups
sanger-tol/genomenote	v1.0	https://github.com/sanger-tol/genomenote
sanger-tol/readmapping	1.1.0	https://github.com/sanger-tol/readmapping/tree/1.1.0
YaHS	1.2a	https://github.com/c-zhou/yahs

### Genome annotation

The BRAKER2 pipeline ( Brůna *et al.*, 2021) was used in the default protein mode to generate annotation for the *Conops quadrifasciatus* assembly (GCA_949752815.1) in Ensembl Rapid Release at the EBI.

### Wellcome Sanger Institute – Legal and Governance

The materials that have contributed to this genome note have been supplied by a Darwin Tree of Life Partner. The submission of materials by a Darwin Tree of Life Partner is subject to the **‘Darwin Tree of Life Project Sampling Code of Practice’**, which can be found in full on the Darwin Tree of Life website here. By agreeing with and signing up to the Sampling Code of Practice, the Darwin Tree of Life Partner agrees they will meet the legal and ethical requirements and standards set out within this document in respect of all samples acquired for, and supplied to, the Darwin Tree of Life Project.

Further, the Wellcome Sanger Institute employs a process whereby due diligence is carried out proportionate to the nature of the materials themselves, and the circumstances under which they have been/are to be collected and provided for use. The purpose of this is to address and mitigate any potential legal and/or ethical implications of receipt and use of the materials as part of the research project, and to ensure that in doing so we align with best practice wherever possible. The overarching areas of consideration are:

•     Ethical review of provenance and sourcing of the material

•     Legality of collection, transfer and use (national and international)

Each transfer of samples is further undertaken according to a Research Collaboration Agreement or Material Transfer Agreement entered into by the Darwin Tree of Life Partner, Genome Research Limited (operating as the Wellcome Sanger Institute), and in some circumstances other Darwin Tree of Life collaborators.

## Data Availability

European Nucleotide Archive: *Conops quadrifasciatus*. Accession number PRJEB59143; https://identifiers.org/ena.embl/PRJEB59143 ( Wellcome Sanger Institute, 2023). The genome sequence is released openly for reuse. The *Conops quadrifasciatus* genome sequencing initiative is part of the Darwin Tree of Life (DToL) project. All raw sequence data and the assembly have been deposited in INSDC databases. Raw data and assembly accession identifiers are reported in Table 1.

## Author information

Members of the University of Oxford and Wytham Woods Genome Acquisition Lab are listed here: https://doi.org/10.5281/zenodo.7125292.

Members of the Darwin Tree of Life Barcoding collective are listed here: https://doi.org/10.5281/zenodo.4893703.

Members of the Wellcome Sanger Institute Tree of Life Management, Samples and Laboratory team are listed here: https://doi.org/10.5281/zenodo.10066175.

Members of Wellcome Sanger Institute Scientific Operations: Sequencing Operations are listed here: https://doi.org/10.5281/zenodo.10043364.

Members of the Wellcome Sanger Institute Tree of Life Core Informatics team are listed here: https://doi.org/10.5281/zenodo.10066637.

Members of the Tree of Life Core Informatics collective are listed here: https://doi.org/10.5281/zenodo.5013541.

Members of the Darwin Tree of Life Consortium are listed here: https://doi.org/10.5281/zenodo.4783558.
