# Supplementary material for: The genome sequence of the Four-banded Bee-grabber, Conops quadrifasciatus De Geer, 1776
Source: Wellcome Open Res. 2024 Mar 8;9:136. [Version 1] doi: 10.12688/wellcomeopenres.21106.1 (PMC11112309; doi:10.12688/wellcomeopenres.21106.1)
Supplement: Peer review history

# Reviewer report: Reviewer response for version 1

**DOI:** 10.21956/wellcomeopenres.23346.r81683

**Published:** 2024-05-27

**Authors:** Joel Gibson

**Affiliations:** 1 Royal British Columbia Museum, Victoria, British Columbia, Canada

This article presents details of the creation of a complete genome assembly for *Conops quadrifasciatus*. This is an excellent choice for a Palearctic representative of the family Conopidae. The family itself is understudied in both systematics and ecology. The database was soundly produced and the annotated genome assembly will be of immense use to those seeking to include Acalyptrate Diptera exemplars in the phylogenomic research. The methods, protocols, and data as presented in the article are entirely appropriate and appear to be replicable and accessible.  

 The background information on Conopidae does appear to be missing some key recent works (e.g., Gibson et al. 2010, 2013). This unfortunately leads to the misnaming of a key morphological structure. Namely, "theca" is an outdated term, replaced by "ventral genital plate".

Are sufficient details of methods and materials provided to allow replication by others?

Yes

Is the rationale for creating the dataset(s) clearly described?

Yes

Are the datasets clearly presented in a useable and accessible format?

Yes

Are the protocols appropriate and is the work technically sound?

Yes

Reviewer Expertise:

Entomology, morphological systematics, molecular systematics, taxonomy, Diptera, ecology, Acalyptratae, shoreline habitats

I confirm that I have read this submission and believe that I have an appropriate level of expertise to confirm that it is of an acceptable scientific standard.

## References

[1] : Phylogeny and taxonomic revision of all genera of Conopidae (Diptera) based on morphological data. *Zoological Journal of the Linnean Society* .2013;167(1) : 10.1111/j.1096-3642.2012.00873.x 43-81 10.1111/j.1096-3642.2012.00873.x

[2] : Placement of Conopidae (Diptera) within Schizophora based on mtDNA and nrDNA gene regions. *Mol Phylogenet Evol* .2010;56(1) : 10.1016/j.ympev.2010.03.026 91-103 10.1016/j.ympev.2010.03.026 20362064

# Reviewer report: Reviewer response for version 1

**DOI:** 10.21956/wellcomeopenres.23346.r79722

**Published:** 2024-05-22

**Authors:** Halide Nihal Açıkgöz

**Affiliations:** 1 Ankara University,, Ankara, Turkey

The manuscript discussed the genome sequence of the *Conops quadrifasciatus* species in detail. This has provided important information about the biology and genetics of the species. The manuscript makes an original contribution, especially regarding genome sequencing.

 Genomic analysis of taxonomically complex groups such as *Conops quadrifasciatus* can help better understand and classify these groups. The manuscript enables comparison of the genome of this species with other Diptera species, allowing to identify genetic differences within and between species.

 The methods used are appropriate and meticulous.

 The results are important so that other researchers can reuse and verify them.

 The manuscript is generally well written and of scientific value. It should be indexed.

Are sufficient details of methods and materials provided to allow replication by others?

Yes

Is the rationale for creating the dataset(s) clearly described?

Yes

Are the datasets clearly presented in a useable and accessible format?

Yes

Are the protocols appropriate and is the work technically sound?

Yes

Reviewer Expertise:

Forensic entomology

I confirm that I have read this submission and believe that I have an appropriate level of expertise to confirm that it is of an acceptable scientific standard.

# Reviewer report: Reviewer response for version 1

**DOI:** 10.21956/wellcomeopenres.23346.r79731

**Published:** 2024-05-02

**Authors:** Sam Heraghty

**Affiliations:** 1 The University of Alabama, Tuscaloosa, Alabama, USA

Summary:

 This article provides a brief summary of the natural history of the dipteran *Conops quadrifasciatus*, a parasitoid of bumble bees and describes the assembly and annotation of its genome. The genome was assembled from the male sample using Hi-Fi sequencing from PacBio as well as Hi-C scaffolding data generated from the female sample. The female sample was also used for RNA sequencing from the head and thorax with the RNA data being used in the annotation pipeline. The produced genome is highly complete with 99% of the assembly organized into 5 autosomes, 2 sex chromosomes and the mitochondrial genome.

 Comments:

 Background:

 In the final section of the background, the authors mention that taxonomy within conopids is complex and this genome will help to resolve uncertainties. It would be useful for the reader if the authors expanded more on this point. Doesn’t need to be anything in depth, could just be a couple of quick lines to give a little more insight into what the taxonomic issues in this group are.

 Genome Sequence Report:

 In the second line of the genome sequence report, the coordinates where the sample was collected are reported in paratheses. Authors should add units or label latitude and longitude as is done in the methods section. Since this is the first place sampling coordinates is reported, readers could be confused.

 Methods:

 Why was Hi-C sequencing done on both the head and thorax of the female sample? If the Hi-C data was only used for scaffolding and to help identify the Y chromosome, shouldn’t Hi-C data only be needed from one of the tissue types?

 In the first paragraph of the methods section

 “ A male specimen was used for DNA sequencing, and a female specimen was used for Hi-C sequencing. A female specimen, used for Hi-C and RNA sequencing (specimen ID Ox001841, ToLID idConQuad2), was netted at the same location on 2021-08-21.”

 The order of the above writing is somewhat confusing. The first sentence could be omitted to make things clearer.

 Also in regard to the above section, Hi-C sequencing is still ultimately DNA sequencing. It would be a little clearer to say the male sample was used for Pacbio sequencing to differentiate how male and female DNA sequencing was different.

Are sufficient details of methods and materials provided to allow replication by others?

Yes

Is the rationale for creating the dataset(s) clearly described?

Yes

Are the datasets clearly presented in a useable and accessible format?

Yes

Are the protocols appropriate and is the work technically sound?

Yes

Reviewer Expertise:

bioinformatics, population genomics

I confirm that I have read this submission and believe that I have an appropriate level of expertise to confirm that it is of an acceptable scientific standard.
